# Early Compaction Might Be a Parameter to Determine Good Quality Embryos and Day of Embryo Transfer in Patients Undergoing Intracytoplasmic Sperm Injection

**DOI:** 10.7759/cureus.23593

**Published:** 2022-03-28

**Authors:** Senem Aslan Öztürk, Mehmet Cincik, Yaprak Donmez Cakil, Sena Sayan, Belgin Selam

**Affiliations:** 1 Clinical Embryology, Institute of Graduate School, Maltepe University, Istanbul, TUR; 2 Department of Histology and Embryology, Maltepe University Faculty of Medicine, Istanbul, TUR; 3 Department of Obstetrics and Gynecology, Cemil Tascioglu City Hospital, Istanbul, TUR; 4 Department of Obstetrics and Gynecology, School of Medicine, Acibadem Mehmet Ali Aydinlar University, Istanbul, TUR; 5 Unit of Assisted Reproductive Technology (ART), Acibadem Altunizade Hospital, Istanbul, TUR

**Keywords:** pregnancy, live birth, infertility, embryo transfer, early compaction

## Abstract

Introduction: Compaction is the first event in embryo morphogenesis. Blastocyst transfer on day five or six has been widely performed in the last decade. We investigated the clinical value of early compaction on day three for evaluation of the transferred embryo quality and pregnancy.

Methods: Four hundred patients with female factor infertility and 776 fresh embryo transfers were included. Two groups were formed: Early compaction group had embryo transfer with at least one day-three embryo exhibiting early compaction. Transferred embryos without early compaction comprised the control group. Embryo transfer was performed on day three or five after the assessment of embryo compaction by a time-lapse technology system. Each patient underwent only a single cycle of embryo transfer. We analyzed fertilization, pregnancy, and live birth rates.

Results: We detected significantly higher numbers of the retrieved oocytes, metaphase II (MII) oocytes, and fertilized oocytes in the early compaction group. Moreover, the transfer of the early compacting embryos on day three resulted in higher pregnancy and live birth rates.

Conclusion: Our data suggest that early compaction might be a factor to determine good quality embryos and embryo transfer day.

## Introduction

The primary aim of assisted reproductive technology (ART) laboratories is the identification of the best embryo with the highest implantation potential to achieve pregnancy. Transfer of a single high-quality embryo prevents multiple pregnancy which is a common treatment‐related adverse outcome of ART associated with significant maternal, fetal and neonatal risks [1]. Embryo transfer is performed either at the cleavage stage on days two to three after fertilization, or at the blastocyst stage on days five to six depending on the stage of embryonic development [2]. Accurate selection of embryos for transfer requires the implementation of a good embryo scoring system for obtaining successful ART outcomes [3]. Typically, evaluation of a cleavage stage embryo includes the assessment of the fragmentation rate, irregularities in blastomeres, multinucleation and the blastomere number [4]. Over the last decade, transferring blastocysts on day five to six became routine in most ART centers, particularly in patients with good prognosis [3,5]. The blastocyst scoring system introduced by Gardner and Schoolcraft in 1999 evaluates the degree of blastocyst expansion, the morphological appearance of the inner cell mass (ICM) and the trophectoderm cells [6].

Compaction, the first event in embryo morphogenesis, usually takes place on day four after fertilization when the embryo proceeds to the morula stage [7-9]. This process is characterized by cell flattening, extensive intercellular connections and polarization to establish the trophectoderm, inner cell mass lineages and the next stages of development [10]. Initiation of compaction is observed at or just after the eight-cell stage in nearly 90% of the embryos analyzed, while the remaining embryos start compaction before the eight-cell stage [11]. There is lack of data in the literature regarding the predictive value of early compaction for embryo evaluation. Several studies propose compaction at day three as a parameter for a good quality embryo [2,7,12]. Early completion of compaction and blastocyst formation are related to higher embryo quality following blastocyst transfer [13,14]. On the other hand, previous research related compaction before the eight-cell stage with aberrant embryonic development [11].

The clinical introduction of the time-lapse technology (TLT) systems enabled ART centers to carefully observe and evaluate the embryo morphology continuously in an uninterrupted culture environment [15]. This study aims to investigate the effects of early embryonic compaction on day three on the rates of pregnancy and live birth retrospectively. Here, the embryo transfer was performed on day three or five following the assessment of embryo compaction by a TLT system, and the pregnancy outcomes were evaluated.

## Materials and methods

Patients and study design

The data of 400 patients who underwent intracytoplasmic sperm injection (ICSI) in Altunizade Acıbadem IVF center between 01.03.2012 and 01.03.2019 were retrospectively analyzed. The ethics board of Maltepe University, Istanbul approved this study (issue number 2019/07-12). Four hundred patients with female factor infertility and 776 fresh embryo transfers were included in the study. A maximum of two compact embryos were transferred. Each patient underwent only a single cycle of embryo transfer. The exclusion criteria were (i) age over 40 years (ii) less than three oocytes retrieved (iii) body mass index (BMI) over 37 kg/m2 and (iv) presence of ovarian hyperstimulation syndrome during gonadotropin hormone-releasing hormone (GnRH) antagonist protocol.

Ovarian stimulation and ICSI

Follicular development was achieved by applying the GnRH antagonist protocol for controlled ovarian stimulation on the third day of the menstrual cycle. Recombinant follicle-stimulating hormone (FSH) (Gonal-F, follitropin alpha, 900IU/1.5 ml; Merck Serono, Lyon, France) or follitropin beta (Puregon, 833IU/ml; MSD, München, Germany) and GnRH antagonist Cetrotide (260-270 mcg cetrorelix acetate; Merck) were used. Monitoring by ultrasound was performed. When the follicles reached around 18-20mm in diameter, recombinant human chorionic gonadotropin (hCG) (Ovitrelle 250mg/0.5ml choriogonadotropin-alpha; Merck Serono, Rome, Italy) was administered. Ovum pick-up (OPU) was performed with transvaginal USG after 34-36 hours. Retrivied oocytes were transferred to medium (G-Mops Plus; Vitrolife, Västra Frölunda, Sweden). Semen sample was prepared by density gradient method to be used in ICSI procedure. ICSI method was applied to appropriate denudated oocytes (MII oocytes). Fertilization control was performed 16-18 hours after the microinjection procedure. All embryos were monitored by a time‐lapse system for five days (EmbryoScope™ Time-lapse System; Unisense FertiliTech, Aarhus, Denmark) Embryo scoring was performed on days three and five. Embryos that displayed compaction on day three were called early compaction embryos.

Patients who met the inclusion criteria were divided into two groups depending on the embryo compaction status on day three. Early compaction group had embryo transfer with at least one day-three embryo exhibiting early compaction. Transferred embryos without early compaction consisted of the control group. Embryo transfers were performed on day three or five depending on the number and quality of the embryos. Maternal serum beta-hCG (β-hCG) levels were measured 10-12 days after embryo transfer to detect pregnancy. Pregnancy was defined as serum β-hCG levels ≥10 mIU/ml. Live birth was defined as at least one infant born after 24 weeks of gestation and surviving for one month. Age, BMI, causes of infertility, number of retrieved oocytes, MII oocytes, fertilized oocytes per cycle and number of cycles, pregnancy and live birth rates for early compaction and control groups were compared.

Statistical analysis

The statistical analysis was performed using SPSS software version 22.0 (IBM Corp., Armonk, NY, USA). Descriptive statistical methods (mean, standard deviation, frequency, ratio, median, minimum, maximum, percentage) were used to examine the data. Kolmogorov-Smirnov test was performed for assessment of normality. Quantitative data such as age, BMI, number of retrieved oocytes, number of MII oocytes, fertilized oocytes per cycle and number of cycles were compared using the Mann-Whitney U test. Pearson Chi-Square test was used to compare qualitative data such as causes of infertility, pregnancy and live birth rates. Statistical significance was defined as a p-value of less than 0.05.

## Results

Four hundred patients in the study were divided into two groups according to the embryo compaction status on day three. Early compaction group had embryo transfer with at least one day-three embryo exhibiting early compaction and corresponded to nearly 50% of all patients and embryo transfers. Transferred embryos without early compaction were evaluated in the control group. The number of patients and embryos depending on the compaction status and transfer day are depicted in Table 1. A total of 776 embryo transfers on days three or five with a maximum of two compact embryos were performed. The patients underwent a single cycle of embryo transfer and therefore only outcome after a single transfer in each patient was included. Three hundred eighty-nine transfers constituted the early compaction group. Non-early compaction group (control group) contained 387 embryos with compaction on day four after fertilization. The embryos with delayed compaction at day five or later were discarded.

**Table 1:** The number of patients and embryos depending on the compaction status and transfer day.

		No. of patients (n:400, %)	No. of embryos transferred (n:776, %)
Day 3 transfers (n,%)	Early compaction group	95 (%23.8)	186 (%24.0)
	Control group	95 (%23.8)	189 (%24.4)
	Total	190 (%47.6)	375 (%48.4)
Day 5 transfers (n,%)	Early compaction group	105 (%26.2)	203 (%26.1)
	Control group	105 (%26.2)	198 (%25.5)
	Total	210 (%52.4)	401 (%51.6)

Total numbers of retrieved oocytes, MII oocytes and fertilized oocytes per cycle were 4422, 3294 and 2565, respectively. The age (32.25±4.58 vs. 31.93±4.34) and BMI (23.41±3.94 vs. 23.54±4.02) of patients and number of cycles (1.68±1.24 vs. 1.71±1.68) were statistically similar between the control and early compaction groups (Table 2). We detected significantly higher numbers of the retrieved oocytes (11.53±5.01 vs. 10.59±4.44) MII oocytes (8.81±3.74 vs. 7.67±3.33) and fertilized oocytes per cycle (7.00±3.51 vs. 5.81±2.88) in early compaction group in comparison to the control group.

**Table 2:** Clinical characteristics of control and early compaction groups. Age, BMI, number of retrieved oocytes, number of MII oocytes, fertilized oocytes per cycle and number of cycles were compared for significance using the Mann-Whitney U test. MII: metaphase II

		Min.-Max (Median)	Mean± SD	p
Age (years)	Early compaction group	20-39 (32)	31.93±4.34	0.474
	Control group	18-39 (33)	32.25±4.58	
BMI (kg/m²)	Early compaction group	17.30-36.29 (22.62)	23.54±4.02	0.855
	Control group	15.57-34.29 (22.54)	23.41±3.94	
Number of retrieved oocytes	Early compaction group	4-40 (12)	11.53±5.01	0.036*
	Control group	4-25 (10)	10.59±4.44	
M II oocytes per cycle	Early compaction group	2-19 (9)	8.81±3.74	0.01*
	Control group	2-20 (7)	7.67±3.33	
Fertilized oocytes per cycle	Early compaction group	1-18 (7)	7.00±3.51	0.00035*
	Control group	1-17 (5)	5.81±2.88	
Number of cycles	Early compaction group	1-8 (1)	1.71±1.68	0.601
	Control group	1-7 (1)	1.68±1.24	

Clinical indications for ICSI treatment included unexplained infertility (nearly 50% of patients in both groups), endometriosis, tubal factor infertility, polycystic ovaries, diminished ovarian reserve, anovulation and multifactorial female infertility. Early compaction and control groups were statistically similar with respect to the cause of infertility (Table 3).

**Table 3:** Causes of infertility in control and early compaction groups. Pearson's Chi-squared test was used to analyze the differences for significance.

	Early Compaction Group (n=200)	Control Group (n=200)	P
Unexplained	94 (%47)	105 (%52.5)	0.380
Endometriosis	31 (%15.5)	28 (%14)	
Tubal Factor	33 (%16.5)	25 (%12.5)	
Polycystic Ovaries	21 (%10.5)	15 (%7.5)	
Diminished Ovarian Reserve	11 (%5.5)	16 (%8)	
Anovulation	4 (%2)	2 (%1)	
Multifactorial Female Infertility	6 (%3)	9 (%4.5)	

We analyzed the relationship between early compaction and pregnancy depending on the day of the embryo transfer (Table 4). Transfer of at least one embryo exhibiting early compaction on day three resulted in a higher pregnancy rate in comparison to the control group (68.8% vs. 58.7%). On the other hand, no significant difference was observed for pregnancy rate when the embryo transfer was performed on day five (79.3% in control group vs. 77.3% in early compaction group). Similarly, both groups had statistically similar pregnancy rates when the overall embryo transfer was considered (69.3% in control group vs. 73.3% in early compaction group).

**Table 4:** Pregnancy rates of the patients depending on the compaction status and transfer day of the embryo (positive βhCG meaning serum βhCG≥10 mIU/ml). Pearson's Chi-squared test was used to analyze the differences for significance. βhCG: beta human chorionic gonadotropin

	Early compaction group	Control group	p
Positive βhCG following 3. day embryo transfer (n, %)	128 (%68.8)	111 (%58.7)	0.042*
Positive βhCG following 5. day embryo transfer (n, %)	157 (%77.3)	157 (%79.3)	0.635
Positive βhCG (n, %)	285 (%73.3)	268 (%69.3)	0.217

Finally, we evaluated the effect of early compaction on pregnancy outcome with respect to the embryo transfer day (Table 5). We demonstrated significantly higher live birth rate in early compaction group when compared to the control group following embryo transfer on day three (30.6% vs. 20.6%). Day five transfers resulted in statistically similar live birth rates (23.7% in control group vs. 30% in early compaction group). Overall live birth rate including transfers on both days was observed significantly higher in early compaction group compared to the control group (30.3% vs. 22.2%, respectively).

**Table 5:** Live birth rates of the patients depending on the compaction status and transfer day of the embryo. Pearson's Chi-squared test was used to analyze the differences for significance.

	Early compaction group	Control group	p
Live birth following 3. day embryo transfer (n, %)	57 (out of 186) (%30.6)	39 (of 189) (%20.6)	0.026*
Live birth following 5. day embryo transfer (n, %)	61 (out of 203) (%30.0)	47 (of 198) (%23.7)	0.154
Live birth (n, %)	118 (out of 389) (%30.3)	86 (of 387) (%22.2)	0.01*

## Discussion

Over the last decade, most ART centers prefer blastocyst-stage transfer approach on days five or six rather than cleavage-embryo transfer on days two or three [3,5]. Transferring the embryo at the blastocyst stage is thought to provide a more analogous timing of exposure to the uterine environment. Moreover, genomic activation on day three allows self-selection hence enabling identification of the embryos with the highest implantation potential [16,9]. On the other hand, extending the duration of culture in vitro may result in exposure to constant stress and suboptimal culture conditions can force the embryo to undergo adaptations [17]. The clinical and laboratory procedures related to the culture to the blastocyst stage are also associated with higher costs and higher degree of discomfort to the patient and to the laboratory staff [18]. Much published data reported better clinical outcomes following blastocyst transfer than cleavage-stage embryo transfer. Several meta-analysis conducted in the recent years demonstrated significantly improved clinical pregnancy and live birth rates after fresh IVF/ICSI transfer with blastocyst compared to cleavage-stage embryos [19,20]. Conversely, another meta-analysis reported no significant differences when the clinical outcomes such as live birth/ongoing pregnancy, clinical pregnancy, miscarriage or cumulative pregnancy were compared, though the quality of the evidence was low in this study [16]. Interestingly, two other meta-analysis identified a higher risk of preterm birth in IVF singleton pregnancies following blastocyst versus cleavage-stage embryo transfer [21,22]. Similarly, several other studies related fresh blastocyst transfer to higher risk of placental and perinatal complications [23-25]. However, a recent retrospective analysis of 67,147 IVF/ICSI cycles found no increase in risks of preterm birth after fresh blastocyst transfer. Furthermore, the babies have similar chances of being healthy after blastocyst and cleavage-stage embryo transfer [26]. Due to the aforementioned reasons, many centers hesitate to apply a general policy for all patients.

Morula, and the associated process of compaction has received little attention compared to other stages of preimplantation development. Day-four embryos are in the process of, and have often completed, the transition from the cleavage stages to the compacted morula stage [9]. Several studies analyzed the initiation of compaction in human embryos in vitro. Embryos that initiated compaction before the eight-cell stage were associated with cytokinetic failure and aberrant embryonic development [11]. Morulas with delayed and/or incomplete compaction were reported to develop less frequently into morphologically optimal blastocysts [27]. A recent retrospective study examining 2,059 morula stage embryos reported pronounced developmental delay at post-compaction stages associated with partial compaction, affecting blastocyst implantation and live birth rates [28].

TLT enables the embryologists to evaluate embryo development dynamically in an uninterrupted culture environment. Embryos are expected to reach certain developmental stages at specific time intervals. The embryos developing into compacted morulae within 94.9 hours and forming regular blastocysts within 113.9 hours exhibited higher pregnancy rates following ICSI [13]. Embryos that completed the first division within 25.90 hours, the second division within 37.88 hours and compaction within 79.93 hours before reaching the blastocyst stage were reported to have a higher implantation potential [14].

We examined a total of 776 embryo transfers, where the early compaction group had at least one day-three embryo exhibiting early compaction and control group with compaction on day four, in the current study. The patients exhibited similar age, BMI, cycle number and clinical indications for ICSI treatment. We observed significantly higher numbers of the retrieved oocytes, MII oocytes and fertilized oocytes per cycle in early compaction group in comparison to the control group. Importantly, we obtained higher pregnancy and live birth rates following embryo transfer on day three in early compaction group. However clinical outcomes after transfer on day five were similar between the two groups. Because of the prominent difference in live birth rates after embryo transfer on day three, the overall live birth rates were also higher in early compaction group in comparison to the control group. Early compaction at day three is suggested as a valuable tool in selection of a good quality embryo [2,7,12]. However, the aforementioned studies examined the pregnancy and/or implantation rates as an outcome following the embryo transfer only on day three. In the present study, we demonstrated the relationship between the early compaction and live birth rates in addition to the pregnancy rates. Moreover, the embryo transfers both on days three and five were included in the present analysis after the assessment of embryo compaction by a TLT system.

## Conclusions

Early compaction may be an additional parameter assisting the embryologists to determine both the good quality embryos and the embryo transfer day. Our data suggests the transfer of the early compacting embryos on day three to obtain higher pregnancy and live birth rates. Future prospective, randomized controlled studies on embryo transfers are required to further enlighten the relation of early compaction to the pregnancy outcome depending on the transfer day.

## References

[1] El-Toukhy T, Bhattacharya S, Akande VA (2018). Multiple pregnancies following assisted conception: scientific impact paper no. 22. BJOG.

[2] Le Cruguel S, Ferré-L'Hôtellier V, Morinière C, Lemerle S, Reynier P, Descamps P, May-Panloup P (2013). Early compaction at day 3 may be a useful additional criterion for embryo transfer. J Assist Reprod Genet.

[3] Gardner DK, Balaban B (2016). Assessment of human embryo development using morphological criteria in an era of time-lapse, algorithms and 'OMICS': is looking good still important?. Mol Hum Reprod.

[4] Nasiri N, Eftekhari-Yazdi P (2015). An overview of the available methods for morphological scoring of pre-implantation embryos in in vitro fertilization. Cell J.

[5] De Croo I, De Sutter P, Tilleman K (2020). A stepwise approach to move from a cleavage-stage to a blastocyst-stage transfer policy for all patients in the IVF clinic. Hum Reprod Open.

[6] Hardarson T, Van Landuyt L, Jones G (2012). The blastocyst. Hum Reprod.

[7] Skiadas CC, Jackson KV, Racowsky C (2006). Early compaction on day 3 may be associated with increased implantation potential. Fertil Steril.

[8] Nikas G, Ao A, Winston RM, Handyside AH (1996). Compaction and surface polarity in the human embryo in vitro. Biol Reprod.

[9] Coticchio G, Lagalla C, Sturmey R, Pennetta F, Borini A (2019). The enigmatic morula: mechanisms of development, cell fate determination, self-correction and implications for ART. Hum Reprod Update.

[10] Ma M, Zhou L, Guo X (2009). Decreased cofilin1 expression is important for compaction during early mouse embryo development. Biochim Biophys Acta.

[11] Iwata K, Yumoto K, Sugishima M, Mizoguchi C, Kai Y, Iba Y, Mio Y (2014). Analysis of compaction initiation in human embryos by using time-lapse cinematography. J Assist Reprod Genet.

[12] Desai NN, Goldstein J, Rowland DY, Goldfarb JM (2000). Morphological evaluation of human embryos and derivation of an embryo quality scoring system specific for day 3 embryos: a preliminary study. Hum Reprod.

[13] Harada Y, Maeda T, Fukunaga E, Shiba R, Okano S, Kinutani M, Horiuchi T (2020). Selection of high-quality and viable blastocysts based on timing of morula compaction and blastocyst formation. Reprod Med Biol.

[14] Mizobe Y, Ezono Y, Tokunaga M (2017). Selection of human blastocysts with a high implantation potential based on timely compaction. J Assist Reprod Genet.

[15] Apter S, Ebner T, Freour T (2020). Good practice recommendations for the use of time-lapse technology. Hum Reprod Open.

[16] Martins WP, Nastri CO, Rienzi L, van der Poel SZ, Gracia C, Racowsky C (2017). Blastocyst vs cleavage-stage embryo transfer: systematic review and meta-analysis of reproductive outcomes. Ultrasound Obstet Gynecol.

[17] Bronet F, Agudo D (2017). Should we forget about embryos till day 5?. Curr Opin Obstet Gynecol.

[18] Rienzi L, Ubaldi F, Iacobelli M (2005). Significance of morphological attributes of the early embryo. Reprod Biomed Online.

[19] Wang SS, Sun HX (2014). Blastocyst transfer ameliorates live birth rate compared with cleavage-stage embryos transfer in fresh in vitro fertilization or intracytoplasmic sperm injection cycles: reviews and meta-analysis. Yonsei Med J.

[20] Glujovsky D, Farquhar C, Quinteiro Retamar AM, Alvarez Sedo CR, Blake D (2016). Cleavage stage versus blastocyst stage embryo transfer in assisted reproductive technology. Cochrane Database Syst Rev.

[21] Dar S, Lazer T, Shah PS, Librach CL (2014). Neonatal outcomes among singleton births after blastocyst versus cleavage stage embryo transfer: a systematic review and meta-analysis. Hum Reprod Update.

[22] Maheshwari A, Kalampokas T, Davidson J, Bhattacharya S (2013). Obstetric and perinatal outcomes in singleton pregnancies resulting from the transfer of blastocyst-stage versus cleavage-stage embryos generated through in vitro fertilization treatment: a systematic review and meta-analysis. Fertil Steril.

[23] Spangmose AL, Ginström Ernstad E, Malchau S (2020). Obstetric and perinatal risks in 4601 singletons and 884 twins conceived after fresh blastocyst transfers: a Nordic study from the CoNARTaS group. Hum Reprod.

[24] Ginström Ernstad E, Bergh C, Khatibi A, Källén KB, Westlander G, Nilsson S, Wennerholm UB (2016). Neonatal and maternal outcome after blastocyst transfer: a population-based registry study. Am J Obstet Gynecol.

[25] Kalra SK, Ratcliffe SJ, Barnhart KT, Coutifaris C (2012). Extended embryo culture and an increased risk of preterm delivery. Obstet Gynecol.

[26] Marconi N, Raja EA, Bhattacharya S, Maheshwari A (2019). Perinatal outcomes in singleton live births after fresh blastocyst-stage embryo transfer: a retrospective analysis of 67 147 IVF/ICSI cycles. Hum Reprod.

[27] Ivec M, Kovacic B, Vlaisavljevic V (2011). Prediction of human blastocyst development from morulas with delayed and/or incomplete compaction. Fertil Steril.

[28] Coticchio G, Ezoe K, Lagalla C (2021). Perturbations of morphogenesis at the compaction stage affect blastocyst implantation and live birth rates. Hum Reprod.

